# The Vice of Parsimony

**Published:** 1875-02

**Authors:** 


THE VICE OF PARSIMONY.—The writer of a very wise book has said that the right getting, spending, lending, borrowing, and bequeathing of money argue an almost perfect man. Just as there are many like Dr. Johnson, who, in the matter of wine, can abstain, but can't be moderate, so there are very many who cannot reach the golden mean in accumulating money. They must be profuse or niggardly. Of the two, the last is the worst. A famous Protestant prelate has told us that "there is not a vice which more effectually contracts and deadens the feelings, which more completely makes a man's affections centre in himself, and excludes all others from partaking in them, than the desire of accumulating. When the desire has once gotten hold of the heart, it shuts out all other considerations but such as may promote its views. In its zeal for the attainment of its end, it is not delicate in the choice of means. As it closes the heart, so its clouds the understanding. It cannot decide between right and wrong." There is a bitter old epitaph on a miser which runs :

"Here lies one who lived unloved and died unlamented; who denied plenty to himself, assistance to his friends, and relief to the poor; who starved his family, oppressed his neighbors, and plagued himself, to gain what he could not enjoy; at last death, more merciful to him than he was to himself, released him from care and his family from want, and here he lies, with the muck-worm he imitated and with the dirt he loved, in fear of a resurrection, lest his heirs should have spent the money he left behind; having laid up no treasure where moth and rust do not corrupt nor thieves break through and steal."

